# Effect of Duration of Irrigation with Sodium Hypochlorite in Clinical Protocol of MTAD on Removal of Smear Layer and Creating Dentinal Erosion

**DOI:** 10.5681/joddd.2012.017

**Published:** 2012-09-01

**Authors:** Mehrdad Lotfi, Negar Moghaddam, Sepideh Vosoughhosseini, Vahid Zand, Mohammad Ali Saghiri

**Affiliations:** ^1^Research Center for Pharmaceutical Nanotechnology, Tabriz University of Medical Sciences, Tabriz, Iran; ^2^Professor, Department of Endodontics, Faculty of Dentistry, Tabriz University of Medical Sciences, Tabriz, Iran; ^3^Post-graduate Student, Department of Endodontics, Faculty of Dentistry, Tabriz University of Medical Sciences, Tabriz, Iran; ^4^Associate Professor, Department of Oral and Maxillofacial Pathology, Faculty of Dentistry, Tabriz University of Medical Sciences, Tabriz, Iran; ^5^Associate Professor, Department of Endodontics, Faculty of Dentistry, Tabriz University of Medical Sciences, Tabriz, Iran; ^6^Kamal Asgar Reseach Center (KARC), Dental Branch, Islamic Azad University of Tehran, Tehran, Iran

**Keywords:** Dentinal tubules, ethylenediamine tetra-acetic acid, smear layer, sodium hypochlorite

## Abstract

**Background and aims:**

The aim of the present study was to compare 1.3% sodium hypochlorite (NaOCl) in MTAD (mixture of tetracycline isomer, acid, and detergent) for the removal of the smear layer and induction of canal erosion.

**Materials and methods:**

38 maxillary incisors were divided in three experimental groups of 10 and two positive and negative control groups of each 4 teeth, and prepared using rotary files. In test groups, 1.3% NaOCl was used for 5, 10 and 20 minutes during preparation followed by MTAD as the final rinse. In negative control group, 5.25% NaOCl was used for 10 minutes followed by 17% Ethylenediamine Tetra-Acetic Acid (EDTA) as the final rinse. In positive control group, dis-tilled water was used for 10 minutes during preparation and then as the final rinse. The samples were examined under scan-ning electron microscope, and the smear layer and dentinal erosion scores were recorded.

**Results:**

Five and 10 min groups had significant differences with 20 min group (p < 0.05). In apical third, 5 and 10 min groups had also significant differences with 20 min (p < 0.05). In the coronal thirds, when the time of irrigation with 1.3% NaOCl increased from 5 min to 20 min, erosion also increased significantly. However, 5 and 10 min groups had no signifi-cant differences with negative control group.

**Conclusion:**

The use of 1.3% sodium hypochlorite for 5 and 10 minutes in the MTAD protocol removes the smear layer in the coronal and middle thirds but does not induce erosion.

## Introduction

Several studies have shown that the commonly used cleaning and shaping techniques of root canals produce a smear layer composed of organic and inorganic components, which consist of odontoblastic projections, microorganisms and necrotic debris.^1 - 3^ Removal of the smear layer and patency of dentinal tubules have an important role in decreasing the time needed to achieve a disinfecting effect of intracanal medicaments.^4^ The smear layer prevents penetration into and adhesion to the dentinal tubules.^5 - 7^ The organic contents of the smear layer can serve as a source of nutritive for some bacterial species.^8 , 9^ Since the smear layer might be contaminated and can protect the microorganisms within the dental tubules, it is advisable to remove the smear layer from the infected and contaminated root canals and disinfect the entire length of the root canal in the final stages of root canal treatment.^3 , 10^

Ethylenediamine Tetra-Acetic Acid (EDTA) and citric acid have been recommended for the removal of the smear layer.^11^ Since EDTA and citric acid have no antibacterial properties,^11^ lasers (CO_2_ and Er:YAG)^11^ and ultrasound techniques have been introduced for the removal of the smear layer; however, none of these techniques result in complete disinfection of the root canal.^12^

A mixture of tetracycline isomer, acid, and detergent (MTAD) introduced by Torabinejad in 2003 as an irrigation solution, can completely eliminate the smear layer.^12^ MTAD is the first canal irrigation solution which can both remove the smear layer and disinfect the root canal. MTAD is a combination of doxycycline (3%), citric acid (4.25%), and polysorbate 80 (0.5%) and is used as a final rinse after chemical and mechanical debridement of the root canal.^13 - 15^

MTAD is more biocompatible than EDTA,^13^ with a similar dissolving effect on the pulp and dentin tissues.^12 , 16 , 17^ Two studies have shown that there are no significant differences in the capacity to remove the smear layer between MTAD and EDTA.^18 , 19^ Many studies have reported a greater extent of dentinal tubule erosion by EDTA in comparison to MTAD.^12 , 17^

Use of 1.3% sodium hypochlorite (NaOCl) as an initial rinse for 20 minutes during canal shaping and use of MTAD for 5 minutes as a final rinse have been recommended, which is referred to as MTAD clinical protocol.^17^

At present, rotary instruments shape the root canal in less than 10 minutes.^20^ In other words, NaOCl is in contact with the canal walls for almost 5-10 minutes as an initial rinse. The effects of different irrigation durations with NaOCl and final rinse with MTAD on dentinal erosion have not been elucidated. Therefore, the aim of the present study was to evaluate the effect of 1.3% NaOCl as an initial rinse on the root canal walls during the 5-, 10- and 20-minute canal shaping procedures with the use of MTAD for 5 minutes as a final rinse.

## Materials and Methods

In the present study, 38 maxillary incisors, extracted as a result of periodontal disease, with mature apices and straight roots were selected. Teeth with calcified root canals and open apices were excluded from the study.

The teeth were divided into three experimental groups of (each containing 10 teeth) and two positive and negative control groups (each containing 4 teeth).

Then the teeth were cleaned with a periodontal curette and stored in 2% chloramine solution. After access cavity preparation, working length was determined by placing #15 K-file (MANI, Yohara, Japan) through the apical foramen and then subtracting 0.5 cm from the measured length. A piece of wax was placed on the root apex. The canals were shaped using RaCe rotary files (FKG Dentaire, La-Chaux de-Fonds, Switzerland) at 350 rpm in a 1/64 air motor (NSK, Kanuma, Japan) and hand files in a crown-down technique. The sequence used was the following: RaCe 40/.1, followed by a #60 k-file to prepare coronal third, RaCe 35/.08 to prepare middle third and RaCe 25/.06, 30/.06, 35/.06 followed by a #40 k-file to prepare apical third.

In group 1, canal debridement and shaping was carried out for 20 minutes. During canal instrumentation, 10 mL of 1.3% NaOCl solution (Merck, Darmstadt, Germany) was used for irrigation of the root canal. The canals were irrigated with almost 1.2 mL irrigation solution after each file and before the use of the next file. A 5-mL syringe (SOHA, Tehran, Iran) with a 28-guage needle (PRORINSE, Johnson City, USA) was used for irrigation of the canals with NaOCl; the needle penetrated into the root canal up to 2 mm short of the WL.

In groups 2 and 3, the canal debridement, shaping and irrigation procedures were the same as those in group 1; however, the duration of canal instrumentation were 10 and 5 minutes, respectively.

Subsequently, the canals were irrigated with 10 mL of distilled water using a 5-mL syringe and a 28-guage needle which penetrated up to 2 mm short of the WL in order to eliminate the interaction between the initial and final irrigation solutions. Then 5 mL of MTAD (Biopure MTAD, Tulsa Dental, OK, USA) was used as a final rinse according to manufacturer’s instructions.

In the negative control group, canal debridement and shaping were carried out for 10 minutes with the use of 10 mL of 5.25% NaOCl. Irrigation was carried out between each consecutive file. Then the canals were irrigated with 10 mL of distilled water in order to decrease interaction between the initial and final irrigation solutions. Finally, 5 mL of 17% EDTA (Roth International Ltd., Chicago, IL) was used for 5 minutes as a final rinse. In the positive control group, canal debridement and shaping were carried out for 10 minutes with the use of 10 mL of distilled water. Finally, 5 mL of distilled water was used for 5 minutes as a final rinse. After the 5-minute final rinse, the canals in all groups were irrigated with 10 mL of distilled water to neutralize the effect of final irrigation to prevent continuation of their action.

All the procedures were carried out by one operator. However, at the end of the study, three observers who were blinded to the type of the irrigation solutions used, grades the amount of remaining smear layer. After the irrigation procedures, two longitudinal cuts were made on the buccal and lingual aspects of the teeth but care was exercised not to impinge on the root canals. Then the teeth were divided into two segments using a dental cement spatula. Both segments were stored in 10% formalin for 24 hours. The fixed specimens were rinsed three times with a sodium cacodylate buffered solution (0.1 M, pH = 7.2), incubated in osmium tetroxide for 1 h, dehydrated with ascending concentrations of ethyl alcohol (30–100%), and placed in a desiccator for at least 24 h. Each specimen was mounted on an aluminum stub and coated with 25 µm of goldpalladium and examined under a scanning electron microscope. Finally, both halves were visualized under a scanning electron microscope (SEM, Cam Scan, MV2300, Czech) at ×2000 magnification. SEM photomicrographs were provided from the center of coronal, middle and apical thirds. The amount of the smear layer was graded based on the method introduced by Torabinejad as follows:^17^

- Grade I: No smear layer within the root canal; all the dentinal tubules were clean and patent.
- Grade II: Moderate smear layer; no smear layer was observed on the root canals; however, the tubules contained some debris.
- Grade III: Thick smear layer; a thick smear layer covered the canal walls; the orifices of the tubules were not visible.

The extent of the erosion of dentinal tubules, too, was graded based on Torabinejad technique:^16^

- Grade I: No erosion; all the tubules seemed normal in size and appearance.
- Grade II: Moderate erosion; erosion was observed in the peritubular dentin.
- Grade III: Severe erosion; intertubular dentin was severely eroded and the tubules were interconnected.

### Data Analysis

Presence and absence of the smear layer and the extent of the erosion of dentinal tubules were graded based on the method introduced by Torabinejad under SEM. Data were analyzed by Kruskal-Wallis and Mann-Whitney tests. Statistical significance was defined at P < 0.05.

## Results

### The Smear Layer

Table 1 presents means and standard deviations of the smear layer grades in the coronal, middle and apical thirds of the canals.

**Table 1:** Smear layer mean and std. deviation of experimental groups at three sections

Treatment	Coronal	Middle	Apical
Sodium hypochlorite 1.3% 5 min	1.71 (0.77)	1.56 (0.51)	2.59 (0.61)
Sodium hypochlorite 1.3% 10 min	1.45 (0.68)	1.26 (0.65)	2.45 (0.68)
Sodium hypochlorite 1.3% 20 min	1.05 (0.22)	1.15 (0.48)	2.00 (0.72)
Negative control	1.14 (0.37)	1.25 (0.46)	2.25 (0.70)
Positive control	2.43 (0.53)	2.57 (0.53)	2.71 (0.78)

**Figure 1:**
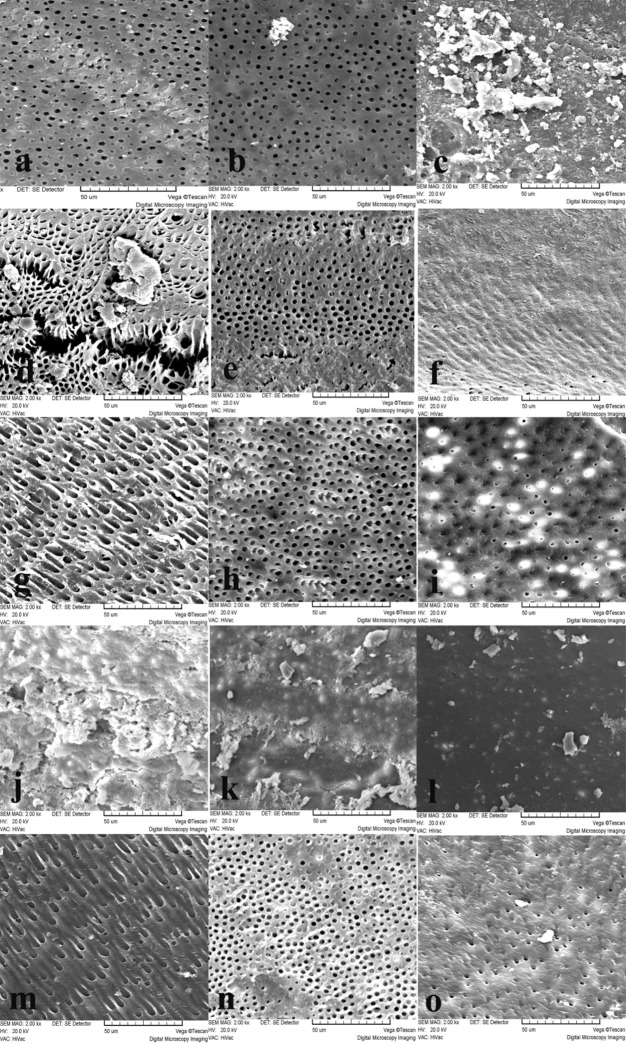
Scanning electron micrographs of coronal (a), middle (b), and apical (c) portions of sodium hypochlorite 1.3% 5 min, coronal (d), middle (e), and apical (f) portions of sodium hypochlorite 1.3% 10 min, and coronal (g), middle (h), and apical (i) portions of sodium hypochlorite 1.3% 20 min, coronal (j), middle (k), and apical (l) portions of positive control and coronal (m), middle (n), and apical (o) portions of negative control.

There were significant differences in the coronal thirds between the experimental groups (P < 0.05). No significant differences were observed between 5 and 10 min groups (P = 0.27). However, both 5 and 10 min groups had significant differences with 20 min group (P < 0.05). There were significant differences in the middle thirds between the experimental groups (P < 0.009). There were significant differences between both 5 and 10 min groups, and both 5 and 20 min (P < 0.05). However, there was no significant difference between 10 and 20 min groups (P > 0.05). In apical third, there was no significant difference between 5 and 10 min groups (P = 0.52), but these both groups had significant differences with 20 min group (P < 0.05). However, there were not any significant differences between experimental and negative control groups (P > 0.05) (Figure 1).

###  Erosion

Table 2 presents means and standard deviations of erosion grades in the coronal, middle and apical thirds in the groups under study.

**Table 2:** Erosion mean and std. deviation of experimental groups at three sections

Treatment	Coronal	Middle	Apical
Sodium hypochlorite 1.3% 5 min	1.39 (0.60)	1.00 (0.00)	—
Sodium hypochlorite 1.3% 10 min	1.95 (0.60)	1.70 (0.73)	—
Sodium hypochlorite 1.3% 20 min	2.55 (0.51)	2.00 (0.56)	—
Negative control	1.86 (0.69)	1.86 (0.69)	—
Positive control	1.00 (0.00)	1.00 (0.00)	—

There were significant differences among experimental groups in the coronal thirds (P < 0.005). In other words, when the time of irrigation with NaOCl 1.3% increased from 5 min to 20 min, erosion also significantly increased. However, both 5 and 10 min groups had no significant differences with negative control (P > 0.05) and only 20 min group had significantly eroded dentin more than negative control (P = 0.02).

In middle thirds, there were significant differences among experimental groups (P < 0.005). There was no significant difference between 10 and 20 min groups (P = 0.12). In other words, increasing the time of irrigation from 10 to 20 min had no significant effect on erosion in middle part of the canal. However, only 5 min group had significant differences with negative control group and both 10 and 20 min groups had no significant effect on erosion in comparison with negative control group (P < 0.05).

In the majority of the samples the surface of dentin was covered with the smear layer in the apical third and no erosion was observed in the specimens without the smear layer (Figure 1).

## Discussion

In the present study, the smear layer and dentin erosion were evaluated by scanning electron microscopy. A scanning electron microscope is capable of evaluating the morphologic details of prepared root canal surfaces, which has been confirmed by several previous studies.^12^

The aim of the present study was to compare canal irrigation solutions in an attempt to simulate clinical situations. Therefore, the manner applied by Prado et al^21^ was used for the closed apex system, in which the apical ends of the teeth were covered with a layer of wax. The aim of the studies which use open apex systems^12 , 17 , 22^ is to evaluate the effect of irrigation solutions; in such cases it is possible for the apical end of the canals to become cleaner in comparison to the condition of teeth inside the tooth sockets due to the free flow of irrigation solutions from the tooth apex; in such cases the clinical situation is not simulated.^23^ At the end of irrigation procedures, the teeth were sectioned into two halves. In the majority of studies, including the studies by Torabinejad et al^12 , 17^ and Prado et al,^21^ only one half of each tooth has been evaluated. In the present study, similar to a study by Lin Dai et al^22^ both tooth halves were evaluated under a scanning electron microscope at ×2000 magnification to increase the accuracy of the study. Several scanning systems have been used in different studies to determine the amount of the smear layer and the extent of erosion. In studies by Saber and Hashem,^24^ Lin Dai et al,^22^and Prado et al^21^ the amount of the smear layer was evaluated in a manner different from that in the present study.

In studies carried out by Torabinejad et al^12 , 17^ and Magrin et al^25^ the method proposed by Torabinejad et al was used to determine the amount of the smear layer. In study carried out by Torabinejad et al^12 , 17^ and Dadresanfor et al^21^ the method proposed by Torabinejad et al was used to evaluate the extent of dentin erosion.

Torabinejad method was used in the present study to determine the amount of the smear layer and the extent of the erosion of dentinal tubules because this method has been used in previous studies on MTAD protocol and the aim of the present study was to evaluate the effect of duration of irrigation with sodium hypochlorite in the MTAD clinical protocol.^12 , 17^

In a study carried out by Torabinejad et al^17^ on the effect of various concentrations of sodium hypochlorite on the ability of MTAD to remove the smear layer the results achieved in the negative control group and one of the experimental groups were comparable to those in the present study; in that study 5.25% sodium hypochlorite was used as the initial rinse and 17% EDTA was used as the final rinse in the negative control group. In the groups corresponding to the experimental groups in the present study, 1.3% sodium hypochlorite was used as the initial rinse and MTAD was used as the final rinse.

The canals were prepared for 18 to 20 minutes; 10 mL of sodium hypochlorite solution was used in a step-wise manner between the files for canal irrigation. After instrumentation, the canals were irrigated for 2 minutes with 5 mL of the final rinse using a 27-gauge needle. In the EDTA group, no smear layer was observed in the coronal and middle thirds; however, a moderate amount of smear layer was detected in the apical third. In the MTAD group no smear layer was observed in the entire length of the canals. A moderate amount of smear layer was observed in other specimens. In the present study, no difference was observed in the amount of the smear layer in the apical third between the MTAD and EDTA groups. The differences between the results of the two studies might be attributed to the use of the open apex system in that study and the use of closed apex system in the present study. In the study carried out by Torabinejad, EDTA exhibited a moderate to severe extent of erosion in the coronal and middle thirds and mild erosion in the apical third. In the MTAD group, moderate erosion was observed in coronal thirds except for one specimen. No erosion was observed in the other samples. In the present study in the 20-minute-instrumentation MTAD group more erosion was observed in the coronal third and the erosion severity in the middle third was comparable to that in the EDTA group. The discrepancies between the results of the two studies might be attributed to differences in the duration of canal irrigation with the final rinse. In the study carried out by Torabinejad the canals were irrigated for 2 minutes with the final irrigation solution; however, in the present study, the final irrigation solution was used for 5 minutes, based on manufacturer’s instructions. Another possible reason might be the fact that in the present study, canal debridement and shaping in the EDTA group lasted 10 minutes whereas the duration was 20 minutes in the study carried out by Torabinejad.^17^

In a study by Franklin, the microstructure of the smear layer within the root canal was evaluated after irrigation with MTAD. The root canals of single-rooted premolars were prepared for 20 minutes with the use of rotary instruments along with irrigation with 10 mL of 2.6% sodium hypochlorite as an initial irrigation solution; 5 mL of 17% EDTA was used as the final rinse in the negative control group and 5 mL of MTAD was used for 5 minutes in the experimental group as the final rinse. In both EDTA and MTAD groups no smear layer was observed in the coronal, middle and apical thirds of the root canals. In addition, no surface erosion was observed in any of the two groups, which is attributed to the fact that sodium hypochlorite was not used. It should be pointed out that the results of the two studies cannot be very well compared because TEM was used in that study and SEM was used in the present study.

MTAD, as a final rinse in the clinical protocol of MTAD, made more erosion in comparison with 5.25% sodium hypochlorite in the 10-minute root canal instrumentation and 17% EDTA as a final rinse in the coronal third. However, since rotary techniques clean and shape the root canals in a short duration of time, use of 1.3% sodium hypochlorite for 5 and 10 minutes in the MTAD groups, remove the smear layer in the coronal and middle thirds and do not induce so much erosion like 20 minutes group. MTAD and 17% EDTA do not have the ability to clean the apical area in the closed apex system.

It is concluded that the use of 1.3% sodium hypochlorite for 5 and 10 minutes in the MTAD protocol removes the smear layer in the coronal and middle thirds and does not induce any erosion.
